# Vascular Wall-Resident Multipotent Stem Cells of Mesenchymal Nature within the Process of Vascular Remodeling: Cellular Basis, Clinical Relevance, and Implications for Stem Cell Therapy

**DOI:** 10.1155/2016/1905846

**Published:** 2016-01-10

**Authors:** Diana Klein

**Affiliations:** Institute for Cell Biology (Cancer Research), University of Duisburg-Essen, University Hospital, Virchowstrasse 173, North Rhine-Westphalia, 45122 Essen, Germany

## Abstract

Until some years ago, the bone marrow and the endothelial cell compartment lining the vessel lumen (subendothelial space) were thought to be the only sources providing vascular progenitor cells. Now, the vessel wall, in particular, the vascular adventitia, has been established as a niche for different types of stem and progenitor cells with the capacity to differentiate into both vascular and nonvascular cells. Herein, vascular wall-resident multipotent stem cells of mesenchymal nature (VW-MPSCs) have gained importance because of their large range of differentiation in combination with their distribution throughout the postnatal organism which is related to their existence in the adventitial niche, respectively. In general, mesenchymal stem cells, also designated as mesenchymal stromal cells (MSCs), contribute to the maintenance of organ integrity by their ability to replace defunct cells or secrete cytokines locally and thus support repair and healing processes of the affected tissues. This review will focus on the central role of VW-MPSCs within vascular reconstructing processes (vascular remodeling) which are absolute prerequisite to preserve the sensitive relationship between resilience and stability of the vessel wall. Further, a particular advantage for the therapeutic application of VW-MPSCs for improving vascular function or preventing vascular damage will be discussed.

## 1. Introduction

The mesenchyme is an embryonic connective tissue which is derived from the mesoderm (the middle embryonic layer) that harbors mesenchymatous cells which have a high rate of division and the ability to spread and migrate in early embryonic development between the ectodermal and endodermal layers [1]. The mesenchymal stem cells (MSCs) are heterogeneous multipotent stem cells which play a pivotal role in the development of all evolving structures and organs from the mesenchyme during ontogeny. In general, these MSCs are considered to originate in the mesenchyme, but embryonic MSCs have recently been shown to derive also from the neuroepithelium and neural crest [2–5]. However, it remains unclear whether ontogenically distinct MSCs are endowed with specific functions [6, 7]. MSCs commonly differentiate into cells of the mesodermal lineage, such as bone, fat, and cartilage cells, but they also have an endodermic and neuroectodermic differentiation potential [4, 8].

During embryogenesis, the mesenchyme differentiates into hematopoietic and connective tissue, whereas MSCs do not differentiate into hematopoietic cells [2, 9, 10]. In particular, the loose, the firm, and the reticular connective tissue, as well as bone, cartilage, smooth muscle and cardiac muscle, kidney and adrenal gland, the hematopoietic system, and blood and lymph vessels, arise from the mesenchyme [11]. In the adult organism, the embryonic mesenchyme is lacking, but reservoirs of MSCs can be found in almost all tissues that contribute to maintenance of the organ integrity. Adult MSCs are multipotent cells which can give rise to mesenchymal and nonmesenchymal tissues in vitro and in vivo. MSCs are commonly characterized by their ability to adhere on plastic, by the expression of a typical panel of MSC surface markers (CD105^+^, CD73^+^, CD90^+^, CD11b^−^, CD79a^−^, CD19^−^ and human leukocyte antigen (HLA-DR)) and the ability to differentiate into different cell types under specific in vitro differentiating conditions (different mesodermal cell lineages including osteoblasts, chondroblasts, adipocytes, and myocytes) [12, 13].

**Figure 1:**
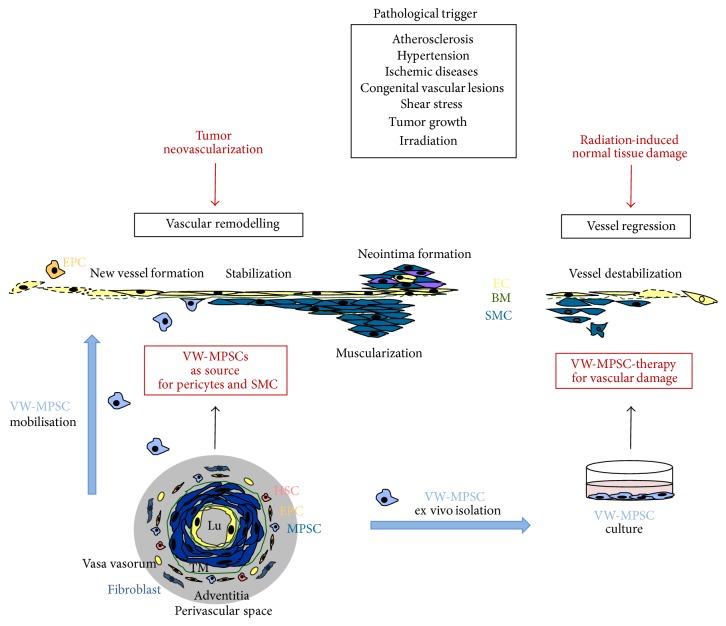
Vascular wall-resident multipotent stem cells of mesenchymal nature within the process of vascular remodeling. Vascular remodeling is a dynamic and strictly regulated process of structural changes, which often occurs as a result of a pathological trigger: atherosclerosis, thrombosis, hypertension, ischemic diseases, congenital vascular lesions, shear stress, irradiation, and tumor growth are crucially characterized by increased vascular remodeling. An ordered remodeling is an absolute prerequisite to preserve the sensitive relationship between resilience and stability of the vessel wall. The association with mural cells (pericytes and smooth muscle cells, SMC) is critical for proper vascular development, stabilization, and maintenance and there is an increasing evidence that these cells originate from multipotent mesenchymal stem cells (MSCs). Intima, media (TM), and adventitia with vasa vasorum are fixed layers of the wall of large arteries and veins. The border between media and adventitia is marked by outer elastic membrane (green). The vasculogenic zone is a vascular mural zone located within the adventitia and close to the tunica media which harbors different subsets of vascular wall stem cells. In particular, vascular wall-resident multipotent stem cells of mesenchymal nature (VW-MPSCs) differentiation into SMC may be induced by tumors, inflammation, and hypoxia in tissue areas around blood vessels contributing to morphogenesis of new vessel walls (e.g., tumor vascularization, intimal lesions, or neointima formation). It is hypothesized that VW-MPSCs are the “first line” cells which were mobilized from their niche towards the tumor and activated to differentiate into pericytes and SMC, which in turn stabilize angiogenic blood vessels, which results in a stabilization and thus normalization of angiogenic tumor blood vessels. In contrast to the direct action of MPSCs during tumor progression by getting mobilized from their niche and subsequent differentiation at the site of injury, the protective effect of exogenous applied MPSCs could also be related to the modulation of paracrine characteristics of these cells. Besides the relatively simple extraction of these cells for autologous transplantation VW-MPSCs might be perfectly suited for a therapeutic application for improving vascular function or preventing vascular damage because tissue-specific stem cells differentiate mainly to the tissue type, from which they derive. HSP: hematopoietic stem cell; EPC: endothelial progenitor cell; light blue: VW-MPSCs yellow, endothelial cells; green basement membrane and elastic membrane; blue, SMC.

The greatest known reservoir of MSCs is the bone marrow, but MSCs reside in many more organs and tissues, such as the adipose tissue, cartilage, muscle, liver and blood, and blood vessels [8, 14–19]. As almost every organ seems to contain MSC, it was suggested that the distribution of MSCs throughout the postnatal organism is related to their existence in a perivascular niche [20]. The existence of a vasculogenic zone has recently been identified in adult human arteries; this particular stem cell niche acts as a source of progenitors for postnatal vasculogenesis [21–24]. A rapidly emerging concept is that the vascular adventitia acts as biological processing center for the retrieval, integration, storage, and release of key regulators of vessel wall function [25, 26]. In response to stress, development of atherosclerotic plaques, or injury, resident adventitial cells can be activated and specified to exhibit different functional and structural behaviors [27–31]. The establishment of a MSC niche in the vascular adventitia provides a basis for the rational design of additional in vivo therapeutic approaches (Figure 1). These findings have implications for understanding MSC biology and for clinical and pharmacological purposes.

## 2. Cellular and Molecular Basis

Vascular remodeling is a dynamic and strictly regulated process of structural changes, which is active in a variety of different physiological processes, such as vessel growth, angiogenesis, and wound healing, after training or during pregnancy. Remodeling often happens in response to a long-term change of hemodynamics, but it also occurs as a result of a pathological trigger: atherosclerosis, thrombosis, hypertension, ischemic diseases, congenital vascular lesions (aneurysms, fibromuscular hyperplasia, and stenosis in collaterals), shear stress, irradiation, and tumor growth are crucially characterized by increased vascular remodeling [32–35]. An ordered remodeling is an absolute prerequisite to preserve the sensitive relationship between resilience and stability of the vessel wall. Herein, the association with mural cells (pericytes and smooth muscle cells, SMC) is critical for proper vascular development, stabilization, and maintenance and there is increasing evidence that these cells originate from multipotent MSCs.

Postnatally, these mural cells have been supposed to be generated by in situ differentiation from local mesenchymal stem or progenitor cells [36]. MSCs can further be induced to differentiate into SMCs via coculturing with SMCs [37]. Moreover, there is evidence for a bone marrow origin of mural cells during adult angiogenesis [38]. In line with this finding, it has been shown that stem cell antigen 1 (Sca1) mesenchymal bone marrow-derived cells are recruited to the site of tumor progression using the RIP-Tag2 model of pancreatic cancer [39]. Besides the bone marrow murine adventitial localized Sca1-positive vascular progenitor cells (AdvSca1) that may play important roles in the maintenance of resident vascular SMC progenitor cells in the artery wall were shown to express transcription factors that are required for SMC differentiation, including serum response factor (SRF) and myocardin family members [40]. Furthermore, it was recently demonstrated that vascular wall-resident CD44-positive multipotent stem cells within the adult human vascular adventitia (VW-MPSCs) are capable of differentiating into pericytes and SMC [15]. A crucial hypothesis concerning these vessel-resident stem cells is that these cells are the “first line” cells, which are available on the basis of their anatomic location as first point of contact for tumor cells and secreted factors [25, 26]. Herein, it was recently shown for the first time that tissue-resident MSCs, which predominantly reside in the adventitia of adult blood vessels and not in the bone marrow are mobilized from their niche, for example, by signals released from tumor cells and contribute to vascular remodeling of tumor blood vessels by differentiating into pericytes as well as SMC [41]. Thus, VW-MPSCs cells are directly involved in vascular remodelling processes in terms of vascular stabilization, serving as a local source for pericytes and SMC (Figure 1).

Processes of new vessel formation are central events in tissue development and repair. In particular, in tumor development, sprouting endothelial cells and/or endothelial progenitor cells form immature blood vessels that lack coverage by pericytes and other mural cells. This first endothelial lining is patchy and angiogenic tumor vessels prove to be functionally inferior. The newly formed vessel cannot actively respond to physiological stimuli because of the lack of smooth muscle elements in their walls [42]. This complicates an efficient administration of intravenous drugs in cancer therapy [43]. Subsequently vascular remodeling takes place, in which association with pericytes and SMC stabilizes these immature vessels resulting in normalization of the vascular structures [42, 44, 45]. Herein, pericytes are recruited upon endothelial platelet-derived growth factor B (PDGF-B) expression to remodel, stabilize, and mature the newly formed vessel. Because pericytes play a central role in tumor angiogenesis and may determine the success of antitumor therapies, a great interest in identifying pericytes in tumor tissue specimens has been developed [46, 47]. The process of vascular remodeling within a tumor is influenced by the therapy with angiogenesis inhibitors [48–50]. Antiangiogenic therapy is characterized by an enhanced vascular remodeling which results in stabilization of the newly formed tumor blood vessels [51–53]. Here, normalization is achieved by the recruitment and integration of mature pericytes in the vessel wall for capillaries as well as SMC for larger vessels which leads to a reduced susceptibility of stabilized vessels to antiangiogenic therapy resulting in an increased therapy resistance [46, 51]. This process is apparently accompanied by enhanced necrosis of tumor tissue [54]. Erber et al. further reported that targeting vascular endothelial growth factor receptors 2 (VEGFR-2) did not result in significant tumor vessel regression due to the pericytes coverage [55]. Targeting both endothelium and pericytes may favor progress in antiangiogenic treatment for tumors because tumor vessels, which are characterized by resistance to antiangiogenic therapy, are characterized by an increase in vessel diameter and a normalization of vascular structures. [56–58].

VW-MPSC specific expression of Nestin (a type VI intermediate filament protein) was recently identified to demonstrate the MSC origin of mural pericytes and SMCs in vascular stabilization processes during tumor progression [41, 59]. Within that scenario, tumor vessels of human colorectal adenocarcinoma metastases under clinical treatment with antiangiogenic therapy were characterized by a prominent stabilization that is achieved by increased integration of Nestin(+) cells into the wall of maturing vessels whereas mature vessels from the tumor surrounding area or healthy tissue were characterized by decreased Nestin expression [41]. In line with these findings, it was shown that the expression of Nestin is specific to developing vascular smooth muscle cells (VSMC) whereas differentiated, postmitotic VSMC are negative for Nestin [60]. Generally, expression of Nestin has been detected in repair processes and in various neoplasms and has been associated with immature and angiogenic blood vessels including proliferating vascular endothelial cells [61]. Even more important, a selective detection of newly formed tumor vessels within cancer tissues using specific markers raises the possibility of molecular targeted therapy via the inhibition of tumor angiogenesis [61]. Thus, the identification of proteins mediating the recruitment of local Nestin-positive MPSCs to angiogenic blood vessels and that of their differentiation into mural cells may allow the definition of new therapeutic targets to counteract pathologic vascular remodeling and to reduce tumor resistance against antiangiogenic drugs.

In future investigations a detailed molecular analysis of VW-MPSCs and their differentiation into pericytes in response to tumor secreted factors may be decisive to elucidate VW-MPSC biology and differentiation. Particularly for cancer therapy, there is an urgent need to identify the signaling molecules which are selectively regulated during the process of new vessel formation and/or subsequent vascular stabilization. Targeting of such molecules might also help to minimize vascular stabilization prompting drug resistance. These investigations shall provide basic knowledge for the design of innovative therapeutic strategies targeting vascular remodeling processes during cancer treatment that are associated with worse prognosis, for example, the generation of drug-resistant tumors, respectively.

## 3. Clinical Relevance and Implications for Stem Cell Therapy

Importantly, tissue-specific stem cells differentiate mainly to the tissue type, from which they derive, indicating that there is a certain code or priming within the cells which is determined by the tissue of origin. Besides the relatively simple extraction of these cells for autologous transplantation, this “priming” might be a particular advantage for the therapeutic application of VW-MPSCs for improving vascular function or preventing vascular damage (Figure 1). The patients vascular wall-resident MSCs can be isolated from small vessel pieces (excess material) which were obtained during surgery (e.g., vena saphena or arteria radialis). Cultivation and ex vivo expansion of these cells would then be performed in order to obtain enough VW-MPSCs for a therapeutic approach.

In various fields of medicine, MSCs have created growing interest due to their unique properties including differentiation and regenerative potential, immune modulation, and migration toward sites of inflammation [62–64]. Stem cells bring new hope for the treatment of many diseases. MSCs are supposed to be one of the most promising types of adult stem cells for cell-based therapies [65–67]. In comparison with embryonic stem cells or induced pluripotent stem cells, MSCs are devoid of the ethical, teratomas formation, and histocompatibility issues. Moreover, MSCs appear to be well tolerated [64, 68]. In general, MSCs exert their therapeutic effects by (1) the ability to home to sites of inflammation after tissue injury and differentiation into various cell types to restore damaged/injured cells, (2) the ability to secrete multiple bioactive molecules capable of stimulating recovery of injured cells and inhibiting inflammation, and (3) the lack of immunogenicity and the ability to perform immunomodulatory functions [69, 70]. Several studies have already shown that MSCs provide an important contribution to tissue neovascularization by migrating to the site of damage and differentiating to restore damaged cell types [71–74]. However, the greatest potential of MSCs in terms of neovascularization is attributed to the trophic effects, for example, to prevent fibrosis and apoptosis or to promote angiogenesis and arteriogenesis due to the production of cytokines for a paracrine action [75–79]. It has already been shown in rats that therapeutically applied MSCs downregulated inflammatory mediators such as TNF-*α*, IL-1*β*, MCP-1, and IL-6 in the lungs, whereas VEGF, VEGFR2, and TGF-1b were upregulated due to paracrine effects and apoptosis of lung cells were reduced. A similar observation could be made in the animal model of pulmonary arterial hypertension. Here, the therapeutic use of bone marrow MSCs led to a reduced vascular remodeling and increased expression of VEGF [80].

While MSCs were initially used for tissue repair and regenerative medicine, discovery of immune-modulating mechanisms of MSCs have prompted their use in immune disorders because MSCs express very low levels of major histocompatibility complex (MHC) Class I antigens and do not express MHC Class II antigens or costimulatory molecules.

MSCs exert an immunosuppressive function in the human body and the molecular mechanisms underlying the MSC-mediated immunosuppression were shown to be species dependent [81–84]. Herein, the the kynurenine pathway which is the main catabolic pathway of the essential amino acid tryptophan has been identified to play a critical role in regulating immune responses [85–87]. In particular, the catabolic enzyme indoleamine 2,3-dioxygenase (IDO) cleaves the aromatic indole ring of tryptophan and therefore initiates the production of several tryptophan degradation products called kynurenines that are known to exert important immunoregulatory functions [81, 86]. MSCs respond to proinflammatory cytokines (e.g., interferon IFN-*γ*) by an increased production of IDO resulting in suppression of the inflammatory response by the MSC-mediated suppression of allogeneic T cell proliferation [87, 88].

Also the release of soluble immunosuppressive factors may play major roles for MSC-mediated immunomodulation. Indeed, MSCs have been shown to express a broad spectrum of secreted molecules, including interferon- (IFN-) *γ*, interleukin- (IL-) 1*β*, IL-6, IL-10, transforming growth factor- (TGF-) *β*1, vascular endothelial growth factor (VEGF), stromal derived factor- (SDF-) 1, HGF, KGF, and prostaglandin PGE2, amongst others [89, 90]. Through the action of these soluble mediators, bone marrow MSCs have been shown to modulate the activation, proliferation, and downstream effects of inflammatory and immune cells in both the innate and adaptive immune systems, including neutrophils, lymphocytes, and macrophages [91–94]. The latter ones infiltrate injured tissues in large numbers and participate in tissue repair. MSCs were capable of reprograming macrophages and thus regulating the immune response. MSCs can regulate the phenotypes in activated macrophages as central mediators of the inflammatory response and preferentially polarize macrophages to the M2 anti-inflammatory phenotype [95–97]. Mechanistically, MSCs constitutively produce IL-6, which polarizes monocytes toward anti-inflammatory IL-10-producing M2 macrophages [98]. This polarization depends on a combination of cell-cell contact mechanisms and the secretion of soluble factors, including IDO and PGE2 [99].

MSCs have further been shown to interact with T- and B-lymphocytes, natural killer cells, and dendritic cells where MSCs suppress T cells, dendritic cell maturation, reduce B-cell activation and proliferation, and inhibit proliferation and cytotoxicity of natural killer cells and promote the generation of regulatory T cells (CD4^+^CD25^+^FoxP3^+^ Treg) via an IL-10 mechanism [64, 100–105].

First clinical applications of stem cell transplantation indicate a potential of these cells in bone regeneration as well as immunosuppressive effects after allogeneic stem cell transplantation [13, 106]. Herein, MSCs isolated from the bone marrow are used in patients with dilated myopathy, cartilage disorders, stroke, and autoimmune diseases [107–109]. Because of their immunomodulatory and engraftment-promoting properties, MSCs have been tested in the clinical setting both to facilitate haematopoietic recovery and to treat steroid-resistant acute graft versus host disease (GVHD), because of their ability to secrete soluble factors capable of stimulating survival and recovery of injured cells and the capacity to home to sites of damage and the ability to blunt exaggerated immune responses [110, 111]. In line with these findings, Zhao et al. reported that bone marrow-derived MSC-based treatment might be able to reduce the incidence and severity of chronic GVHD in acute GVHD patients, mainly by improving thymic function without increasing the risk of infection or tumor relapse [112]. Furthermore, treatment with MSCs isolated from the bone marrow of related donor might represent an effective therapeutic strategy for patients with refractory aplastic anemia [113].

However, for most of the clinical applications of MSC large number of cells for transplantation are required. The possibility of bone marrow recovery in contrast is a very invasive procedure and only 0.01 to 0.001 percent of mononuclear cells in the bone marrow are MSCs, so that other more accessible sources would represent good alternatives [16]. Herein, abundance, easiness of isolation, and proliferative potential may be deciding factors while choosing a source of MSC [114]. Besides, the bone marrow MSCs can be obtained from peripheral blood, human adipose tissue, Wharton's jelly, umbilical cord and umbilical cord blood, amniotic fluid, placenta, fetal liver, lung and dermal tissue, and even dental pulp [115–121].

Above all, cord blood tissue, adipose tissue, and peripheral blood with MSCs released from the bone marrow could be a good and easily accessible source of human MSCs [122–124]. However, the percentage of MSC in peripheral blood is very low. Therefore, the donor must be stimulated with GM-CSF to increase this percentage by a MSC mobilization from the bone marrow, but this is very distressing and stressful for the patient. The amount of MSCs which can be obtained from adipose tissue is promising by yielding approximately 500-fold greater numbers than MSCs from the bone marrow or peripheral blood [125]. Interestingly, in a clinical setting intravenously infused autologous adipose-derived MSC displayed various improvements in tactile sensitivity in patients suffering from spinal cord injury while no serious adverse events were detected after short term follow-up and no tumor development was observed a few years later [126]. As an alternative source, the isolation efficiency from Wharton's jelly MSCs was reported to be even higher [127]. Side-by-side comparison of MSC from bone marrow, adipose tissue, and Wharton's jelly demonstrated that Wharton's jelly-derived MSCs have the highest proliferative capacity among tested cell types [114, 122].

In summary, MSC-based cell therapies are very promising in various clinical fields.

For the reasons listed above, adult human blood vessels or, in detail, vessel-resident MSCs are another promising source of MSCs which could be particularly well suited for a therapeutic application to improve vascular function or prevent vascular damage. Interestingly, very recently, it was shown that the therapeutic application of VW-MPSC was particularly well suited for the radioprotection of endothelial cells in a murine model of radiation-induced lung injury [128]. Herein, intravenously injected GFP-labeled MPSCs derived from mouse aorta into fibrosis-sensitive C57Bl/6 mice at different time points after whole thorax irradiation counteracted radiation-induced vascular damage and reduced metastasis of circulating tumor cells to the irradiated lung tissue. The adoptive transfer of MSCs normalized vascular dysfunction through inhibition of endothelial Mmp2, reversed epithelial cell senescence and certain aspects of the associated senescence-associated secretory pattern (SASP), and reduced the risk of lung metastasis. In contrast to that direct action of MPSCs during tumor progression by getting mobilized from their niche and subsequent differentiation at the site of injury, the protective effect of exogenous the applied MPSCs here was related to the modulation of paracrine characteristics of these cells. Transplantation of MSCs has established itself as a potential strategy for the treatment of lung diseases [90, 129, 130]. There are few ongoing clinical trials with MSCs in chronic lung disease and the extrapolation of these data for future therapeutic applications in patients with idiopathic pulmonary fibrosis [131, 132]. In this regard, numerous studies have shown that lung epithelial or endothelial cells are rarely derived from MPSCs [133, 134]. Therefore, engraftment in the lung as structural epithelium or endothelium is not currently considered the mechanism by which MSCs can repair lung tissue [90]. Instead, previous studies have shown that bone marrow MSCs migrate to injured tissues, communicate with injured parenchyma cells, and function in wound healing through the production of paracrine-soluble cytokines and growth factors which modulate the regeneration of the epithelium and endothelium [89].

## 4. Conclusion

The adventitial zone is the multipotent MSC niche in vivo, where local cues coordinate the transition to progenitor and mature cell phenotypes. Herein, it is proposed that MPSCs stabilize blood vessels and contribute to tissue and immune system homeostasis under physiological conditions and assume a more active role in the repair of focal tissue injury.

The central hypothesis concerning these VW-MPSCs is that these cells are the “first line” cells which were mobilized from their niche towards a pathological trigger followed by migration to the site of damage and differentiation to restore damaged cell types. There is increasing evidence that tissue-resident multipotent stem cells which reside within the vascular adventitia and not circulating multipotent stem cells are the major source for pericytes and SMC in the vascular stabilization processes. Vascular stabilization is crucial for the survival of newly formed vessels, as immature vessels may be subject to regression and cell death quickly when the angiogenic stimulus is removed. However, the greatest potential of MSCs in terms of neovascularization is attributed to the trophic effects which are the production of cytokines for a paracrine action.

Application of adult MSCs is a valuable therapeutic option for the improvement or prevention of several diseases including lung diseases or the regeneration of diseased lung tissue, because these cells are relatively easily available, have immunomodulatory effects and have the capacity for cell differentiation. Generally, MSCs are isolated from bone marrow or fatty tissue. The isolation method is quite simple and the relatively unspecific isolation method might explain certain heterogeneity of isolated cells (different cellular morphologies and cloning growth pattern as well as initial colonies formed). Besides the relatively simple extraction of VW-MPSCs for autologous transplantation, these cells might be perfectly suited for a therapeutic application for improving vascular function or preventing vascular damage because tissue-specific stem cells differentiate mainly to the tissue type from which they derive.

Further investigations shall provide basic knowledge for the design of innovative therapeutic strategies targeting different paradigmatic vascular remodeling processes during cancer treatment that are associated with worse prognosis, for example, the generation of drug-resistant tumors and the induction of normal tissue damage, respectively.
